# Supplementary material for: Protocol for Cerebellar Stimulation for Aphasia Rehabilitation (CeSAR): A randomized, double-blind, sham-controlled trial
Source: PLoS One. 2024 Aug 26;19(8):e0298991. doi: 10.1371/journal.pone.0298991 (PMC11346736; doi:10.1371/journal.pone.0298991)
Supplement: S2 File — (DOCX) [file pone.0298991.s002.docx]

**Johns Hopkins Medicine - eForm A**

- **Use the section headings to write the eForm A, inserting the appropriate material in each. If a section is not applicable, leave heading in and insert N/A.**
- **When submitting eForm A (new or revised), enter the date submitted to the field at the top of eForm A.**

***************************************************************************************************

1. **Abstract**
2. *Provide no more than a one page research abstract briefly stating the problem, the research hypothesis, and the importance of the research.*

In this project, we will investigate the effects of cerebellar transcranial direct current (tDCS) stimulation during language therapy for naming in individuals with chronic aphasia (>6 months post stroke). Naming difficulties are a persistent and common symptom in aphasia after left-hemisphere (LH) stroke. Although the interventions to improve naming can have benefits (e.g., Hillis, 1998; Nickels, 2002; Raymer et al., 2007; Wisenburn & Mahoney, 2009), a massive number of treatment sessions is usually required to show gains, particularly in individuals with chronic, large LH stroke. Transcranial direct cortical stimulation (tDCS) is a promising adjunct to traditional SALT (for reviews see Holland and Crinion, 2012; de Aguiar et al., 2015; Breining and Sebastian, 2020). tDCS is a safe, non-invasive, non-painful electrical stimulation of the brain which modulates cortical excitability by application of weak electrical currents in the form of direct current brain polarization (Weiss & Bikson, 2014). It is usually administered via saline-soaked surface sponge electrodes attached to the scalp and connected to a direct current stimulator with low intensities (Lang et al., 2005). Research paradigms employing tDCS are based on principles of neuroplasticity. Prior investigators have mainly focused on the role of LH in language recovery, wherein the electrode is placed in the left frontal or temporal region (e.g., Baker, Rorden, & Fridriksson, 2010; Fridriksson et al., 2011, 2018). However, left hemisphere lesions common in post-stroke aphasia affect the electrical field in unpredictable ways, potentially preventing stimulation from reaching perilesional tissue associated with optimal recovery

The PI’s prior work addressed this problem by stimulating a novel region, the right cerebellum. The right cerebellum is connected to the left hemisphere and involved in a variety of cognitive and language functions, including naming, which is often impaired in people with aphasia. The PI’s prior work shows that cerebellar tDCS is safe, easily tolerated, and improved language skills in a number of stroke participants with aphasia (Sebastian et al., 2017, 2020). The proposed project will build on these findings by conducting a randomized, double-blind, sham-controlled, trial to determine the effectiveness of cathodal tDCS to the right cerebellum for the treatment of post-stroke aphasia. We will compare the effects of 15 sessions of cerebellar tDCS combined with an evidenced-based anomia treatment (semantic feature analysis, SFA) to 15 sessions of sham combined with SFA treatment in patients with chronic (at least 6 months post stroke) aphasia. We will evaluate the effects of cerebellar tDCS on naming untrained items as well as the effects on functional communication, content, efficiency, and word-retrieval of picture description, and quality of life. This project will also identify imaging and linguistic biomarkers to determine the characteristics of stroke patients that benefit from cerebellar tDCS and SFA treatment. Individual response to tDCS treatment is highly variable, and little is known about how factors related to imaging and linguistic characteristics combine to induce treatment responsiveness. We will carry out resting state functional magnetic resonance imaging (rsfMRI), diffusion tensor imaging (DTI), high resolution structural imaging, and detailed linguistic testing before the start of treatment to determine whether these factors can predict response to cerebellar tDCS and/or SFA. The long-term aim of this study is to provide the basis for a Phase III randomized controlled trial of cerebellar tDCS vs sham with concurrent SLT for treatment of chronic aphasia.

**2. Objectives**

**Primary Objective**: The primary objective of this proposal is to determine whether cathodal right cerebellar tDCS along with SFA can improve untrained naming outcome in participants with chronic aphasia.

**Hypothesis:** 15 sessions of cathodal cerebellar tDCS plus SFA will be associated with greater improvement in naming untrained pictures (as measured by the change in Philadelphia Picture Naming Test, PNT, Roach et al., 1996),1-week post treatment, compared to sham plus SFA.

**Secondary Objectives:**

Secondary Objective 1: To determine whether cathodal right cerebellar tDCS combined with SFA will improve trained naming, discourse, and functional communication skills in participants with chronic aphasia.

**Hypothesis** 1a. 15 sessions of cathodal cerebellar tDCS plus SFA will be associated with greater improvement in naming trained pictures compared to sham plus SFA.

**Hypothesis** 1b. 15 sessions of cathodal cerebellar tDCS plus SFA will result in greater improvement in discourse (as measured by change in total content units (CU) and syllable per CU in picture description) compared to sham plus SFA.

**Hypothesis** 1c. 15 sessions of cathodal cerebellar tDCS plus SFA will result in greater improvement in functional communication skills (as measured by change in Communication Activities of Daily Living–CADL-3, Holland et al., 2018) compared to sham plus SFA.

**Secondary Objective 2**: To determine whether cathodal right cerebellar tDCS combined with SFA will improve 3 outcomes measures from the Research Outcome Measurement in Aphasia-Core Outcome Set (ROMA-COS, Wallace et al., 2018) at baseline, 1-week post treatment, and follow up time points: The Western Aphasia Battery-Revised (Kertesz, 2007) will be administered as a part of the baseline testing. We will also assess changes in emotional well-being (measured by General Health Questionnaire (GHQ, Goldberg & Hillier, 1979) and quality of life (measured by Stroke and Aphasia Quality of Life Scale (SAQOL, Hilari et al., 2003).

**Hypothesis**: 15 sessions of cathodal cerebellar tDCS plus SFA will result in greater improvement on the WAB-R, GHQ, and SAQOL compared to sham plus SFA.

**Secondary Objectives 3:** To identify whether neural (functional and structural) biomarkers and linguistic characteristics can predict response to cerebellar stimulation and SFA treatment.

**Hypothesis**: Baseline resting state functional connectivity, structural connectivity, lesion volume and site, overall aphasia severity, and naming severity will predict response to cerebellar tDCS treatment with SFA or SFA treatment alone. This exploratory aim may identify stroke patients who are mostly likely to benefit from cerebellar tDCS and/or SFA. This result may have significant implications for designing a Phase III randomized controlled trial.

**3. Background** (*briefly describe pre-clinical and clinical data, current experience with procedures, drug or device, and any other relevant information to justify the research)*

Introduction

Aphasia is one of the leading causes of disability following stroke. Anomia or difficulty with naming is the most common deficit in individuals with aphasia. Although the interventions to improve naming can have benefits (Hillis, 1998; Kiran & Thompson, 2003; Nickels, 2002; Raymer et al., 2007; Wisenburn & Mahoney, 2009) a massive number of treatment sessions is usually required to show gains, particularly in individuals with chronic large LH stroke. There is evidence that tDCS may be useful for enhancing the effects of behavioral aphasia treatment. Stimulating the residual left hemisphere region is the most common approach based on the observation that optimal recovery involves the functional re-recruitment of the remaining left-hemisphere tissue (Fridriksson et al., 2012; Hamilton et al., 2011; Hartwigsen & Saur et al., 2019). However, encephalomalacia filled with cerebrospinal fluid at the site of stroke affects the electrical current flow, reducing the exposure of the targeted perilesional tissue to stimulation (Turkeltaub et al., 2016). This issue makes selection of optimal electrode locations in the left hemisphere difficult. Approaches to address this issue involve advanced electrical field modeling methods (e.g., Datta et al., 2011; Dmochowski et al., 2013) or individualized electrode placement based on pre-treatment functional magnetic resonance imaging (fMRI) scans so that stimulation targets residual functional tissue (Baker et al., 2010; Fridriksson et al., 2011, 2018). However, advanced electrical field modeling and fMRI are cost-intensive and require substantial technological expertise. In addition, fMRI scan as a requirement for entry into tDCS treatment studies significantly limits the number of patients that can be treated, because many people have contraindications for MRI or find it difficult to complete tasks inside the scanner, particularly under stressful or timed conditions. All of these factors would limit the incorporation of tDCS into routine speech language pathology clinical practice.

In 2015, we proposed a novel approach to augment aphasia treatment by stimulating the right cerebellum. The right cerebellum is not only involved in cognitive and language functions (for reviews see Desmond & Fiez, 1995; Murdoch, 2010; De Smet et al., 2013), but is also distant enough from typical stroke locations associated with aphasia that electrical current flow patterns are unlikely to be affected by the encephalomalacia (Turkeltaub et al., 2016). In addition, this approach is suitable for patients who have large left hemisphere strokes and aphasia associated with bilateral hemispheric strokes. Our group published the first study showing that cerebellar tDCS has the potential to augment aphasia treatment (Sebastian et al., 2017). Our recent randomized, sham controlled, within-subject crossover trial, found that right cerebellar tDCS combined with computerized aphasia treatment improved picture naming performance for trained and untrained items (Sebastian et al., 2020). However, participants who received cathodal stimulation to the cerebellum showed significantly greater gains in naming (relative to sham) compared to participants who received anodal stimulation, indicating that cathodal stimulation might be more favorable than anodal stimulation to augment aphasia treatment.

tDCS is believed to enhance neural plasticity by temporarily modulating resting membrane potentials of neurons in targeted areas (Nitsche & Paulus, 2001; Brunoni et al., 2012). Anodal stimulation may lead to depolarization of the neuronal membranes resulting in greater excitability, whereas cathodal stimulation may lead to hyperpolarization resulting in lower excitability. Animal and human studies indicate that cerebellar tDCS is most likely to produce its effects by polarizing Purkinje cells - the inhibitory output neurons of the cerebellar cortex - and thereby changing the levels/pattern of activity in the deep cerebellar output nuclei, which are the efferent targets of the Purkinje cells (Galea et al., 2009; Grimaldi et al., 2016). Critically, one of the deep cerebellar nuclei, the dentate nucleus, has a disynaptic excitatory connection through the thalamus to the cortical language areas. Based on this known circuitry, we hypothesize that a single session of right cathodal cerebellar stimulation will result in transient depression of Purkinje cell activity, thereby reducing the inhibitory signals that the cerebellum sends to the cortical language areas. Thus, it is plausible that multiple sessions of cathodal cerebellar tDCS will provide cortical excitation, thereby facilitating the engagement of the residual left hemisphere language areas.

Studies show that while the increase or decrease in excitability induced by tDCS may not be adequate on its own to induce long-term changes, the additional synaptic activation (by co-stimulation via language treatment) may be sufficient to induce long-term plasticity (Rioult-Pedotti et al., 2000; Fritsch et al., 2010). In this proposal, we will combine cerebellar tDCS and semantic feature analysis (SFA) treatment (Boyle, 2010). This study will use SFA treatment (for reviews see Boyle, 2010, Maddy et al., 2014, Oh et al., 2016). SFA is a semantically based treatment approach for naming deficits. SFA was chosen for this study for three main reasons (1), SFA has a strong potential for promoting acquisition and generalization effects for participants with anomia, (2) SFA is an effective therapy for treating naming deficits for individuals with a range of aphasia types and severities, (3) SFA is a treatment that is frequently used by practicing SLPs.

The driving premise of SFA treatment is that when individuals generate semantic features of a target word (i.e., accessing their semantic network), they improve their ability to retrieve the target because they have strengthened access to its conceptual representation (Boyle & Coelho, 1995; Boyle, 2010). The theoretical mechanism by which SFA promotes generalization comes from the spreading activation theory (Collins & Loftus, 1975) which posits that accessing/activating a particular lemma (or its features) results in activation of the lemmas of semantically related concepts.

The PI’s prior work in cerebellar tDCS in aphasia has shown that individual response to tDCS treatment is highly variable. However, little is known about how factors related to imaging and linguistic characteristics combine to induce treatment responsiveness. We will carry out resting state functional magnetic resonance imaging (rsfMRI), diffusion tensor imaging (DTI), high resolution structural imaging, and detailed linguistic testing before the start of treatment to determine whether these factors can predict response to cerebellar tDCS and/or SFA. This exploratory aim may identify stroke patients who are mostly likely to benefit from cerebellar tDCS and/or SFA. This result may have significant implications for designing a Phase III randomized controlled trial.

**4. Study Procedures**

1. *Study design, including the sequence and timing of study procedures*

*(distinguish research procedures from those that are part of routine care).*

Study Procedures Overview

This study, Cerebellar Stimulation for Aphasia Rehabilitation (CeSAR), is a Phase II trial of cathodal cerebellar tDCS plus anomia (SFA) treatment vs. sham plus anomia (SFA) treatment, evaluated in double-blind, randomized, sham-controlled design in chronic stroke. Participants with aphasia will be enrolled at 2 sites within the Johns Hopkins Rehabilitation Network at least 6 months after the onset of stroke. The two sites will be the Johns Hopkins Hospital and Howard County General Hospital. Participants will undergo language testing and MRI to determine eligibility and then will be randomly assigned to cerebellar tDCS plus anomia treatment or sham plus anomia treatment. Participants will receive 15 sessions of anomia treatment (3-5 sessions per week), as in the ongoing K99/R00 study. Participants will be evaluated prior to the start of treatment, one-week post treatment, 1, 3, and 6-months post- treatment on primary and secondary outcome variables.

Participants will be randomized 1:1 (cathodal tDCS or sham tDCS), stratified by site (JHH vs Howard County), aphasia type (fluent vs. non-fluent, classified using WAB-R), and severity (classified using WAB-R Aphasia Quotient in 4 categories (very severe aphasia: 0–25, severe aphasia: 26–50, moderate aphasia: 51–75, and mild aphasia: 76-93.8). All participants, the members of the study team who administered the assessments and treatments as well as the study biostatistician performing the statistical analyses will be blinded.

In order to minimize the need for research-only in-person visits, telemedicine visits will be substituted for portions of clinical trial visits where determined to be appropriate and where determined by the investigator not to increase the participants risks. For the current study, we will utilize telemedicine visits when appropriate for consenting and for all the assessments visits (visits 1- 3, 20-23). Prior to initiating telemedicine for study visits the study team will explain to the participant, what a telemedicine visit entails and confirm that the study participant is in agreement and able to proceed with this method. Telemedicine acknowledgement will be obtained in accordance with the Guidance for Use of Telemedicine in Research. In the event telemedicine is not deemed feasible, the study visit will proceed as an in-person visit. Telemedicine visits will be conducted using HIPAA compliant method approved by the Health System and within licensing restrictions.

Visit 1: During the first visit, participants will sign informed consent and undergo screening assessments. This will include tDCS and MRI safety screening, and aphasia diagnosis

Visit 2 and 3: This involves detailed language testing (see below) including computerized naming assessments. This will be the baseline language testing.

Visit 4: Participants may be asked to get structural MRI (to determine site of lesion) and fMRI (resting state). If the participant does not have a lesion in the right cerebellum, the participant will be able to start intervention. This visit is only for participants who consent to MRI and have no contraindication.

Participants will also undergo neurological exam in visit 1, 2 or 3.

Visit 5-19: This will involve the treatment sessions. Prior to the start of treatment, participants will be randomly assigned to receive sham plus SFA or active tDCS plus SFA.

Visit 20: This visit will involve 1-week post-treatment language testing. Assessment of primary and secondary outcome variables.

Visit 21: This visit will involve 1-month post-treatment language testing. Assessment of primary and secondary outcome variables.

Visit 22: This visit will involve 3-months post-treatment language testing. Assessment of primary and secondary outcome variables.

Visit 23: This visit will involve 6-months post-treatment language testing. Assessment of primary and secondary outcome variables.

Procedures for Visit 1

The following procedures will be performed:

1. Obtain written informed consent: A signed and dated informed consent form will be obtained from each participant before conducting any screening procedures. Participants will then be assigned a temporary identification number for the purposes of initial screening.

All research staff authorized to obtain informed consent will have completed the Miami CITI course in the Responsible Conduct of Research and Protection of Human Subjects prior to their involvement with the study. Furthermore, they will be oriented to the study and trained by the study PI and study co-investigators who have all had extensive training and experience in the ethical and practical aspects of informed consent procedures.

2. Administer the tDCS safety screening and MRI safety screening

3. Review inclusion/exclusion criteria.

4. Obtain medical history.

5. Administer neurological examination

6. Administer aphasia diagnostic test. The Western Aphasia Battery-Revised (WAB-R) will characterize the participants’ overall language impairment through the evaluation of the main clinical aspects of language functioning, including speech content, speech fluency, auditory comprehension, repetition, and naming. The WAB-R allows for the differentiation of these specific language abilities, as well as the classification of aphasia type. The WAB-R also yields a composite score, the Aphasia Quotient, which provides an overall measure of severity, in which lower scores denote more severe aphasia (Kertesz, 2007). Both the aphasia severity and aphasia type will be used to stratify participants for randomization. Participants will be randomized 1:1 (cathodal tDCS or sham tDCS), stratified by site (JHH vs Howard County), aphasia type (fluent vs. non-fluent, classified using WAB-R), and severity (classified using WAB-R Aphasia Quotient in 4 categories (very severe aphasia: 0–25, severe aphasia: 26–50, moderate aphasia: 51–75, and mild aphasia: 76-93.8). Covariate-adaptive randomization method developed by Pocock and Simon, 1975 will be implemented in REDCap. This method ensures balance on important baseline covariates for each treatment arms by calculating the difference in these covariates (site, aphasia type and severity) each time a participant needs be randomized and then randomizes with high probability (80%) to the arm that corrects the imbalance on covariates.

Procedures for language testing (Visits 2 and 3), All assessments will be administered and scored by a Speech Language Pathologist (SLP).

1. **Administer the Boston Naming Test-Second Edition (BNT, Kaplan et al., 2001)**: The BNT represents a measure of object naming abilities from a corpus of 60 line drawings. Object names are ranked along a continuum, with easier, more high frequency words appearing at the beginning of the test and more difficult, lower-frequency words appearing near the end. To eliminate participant frustration, the BNT implements a ceiling effect so that once the participant incorrectly names eight items in a row, testing will cease, with the assumption that (s)he would not correctly name the upcoming, more difficult words (Kaplan et al., 2001). SLPs will refer to the manual for explicit instructions regarding administration and scoring procedures. Administration time will range between 5-20 minutes.

2. **Administer the Apraxia of Speech Rating Scale (Strand et al., 2014**), to rate frequency and severity of particular characteristics of apraxia of speech (AOS): The Apraxia of Speech Rating Scale is a rating scale, in which speech characteristics are evaluated in terms of frequency and severity. Higher scores indicate more severe apraxia of speech. SLPs will refer to the manual for explicit instructions regarding administration and scoring procedures. Administration time will range between 10-15 minutes.

3. **Administer the Pyramids and Palm Trees Test (PPTT) (Short form, Howard & Patterson, 1002; Breining et al., 2015):** The PPTT is a test of semantic processing. This test assesses the degree to which a participant can access meaning from pictures. Information from the test will help determine whether a participant’s difficulty in naming or pointing to a named picture is due to a difficulty in retrieving semantic information from pictures. SLPs will refer to the manual for explicit instructions regarding administration and scoring procedures. Administration time will range between 10-20 minutes.

4. **Administer the Cognitive-Linguistic Quick Test, Helm-Estabrooks, 2001).** This test assesses strengths and weaknesses in five cognitive domains (Attention, Memory, Executive Functions, Language, and Visuospatial Skills). CLQT provides a brief and insightful measure of the status of an individual’s cognitive-linguistic functioning. The CLQT consists of ten tasks: personal facts, symbol cancelation, confrontation naming, clock drawing, story retelling, symbol trails, generative naming, design memory, mazes, and design generation.

5. **Administer Computerized Philadelphia Naming Test (Roach et al., 1996).** This test is administered to determine generalization from trained to untrained items (Primary Outcome Variable). This test is a 175-item picture naming test for the psycholinguistic exploration of lexical access in nonaphasic and aphasic speakers. Items are line-drawn exemplars of animate and inanimate objects (all non-unique, i.e., no famous people or landmarks). Participants whose correct naming score exceeds an average of 140/175 on the PNT baseline sessions will be excluded to leave at least a 20% improvement margin. That is, patients who already score close to ceiling may have limited room for naming improvement as measured by the PNT.

6. **Administered computerized trained item assessment naming task (Secondary outcome Variable)**. This name task is administered to determine whether the participant’s ability to name the trained items improved over the course of treatment. This consists of the 50 pictures that are all nouns.

7. **Administer the Communication Activities of Daily Living (CADL, Holland et al., 2018, secondary outcome variable)**. The CADL-third edition will be used to measure changes in everyday functional communication skills. The CADL was developed to assess communication abilities among people with aphasia in a naturalistic and standardized manner. Tasks require a range of abilities, including the use of numbers, reading, writing, nonverbal communication, and humor appreciation. Responses receive a score of 0, 1, or 2 based on the effectiveness of communication in the different contexts, generally allowing for verbal or nonverbal methods of conveying information.

8. **Administer the BDAE cookie theft picture description (Goodglass et al., 2001, secondary outcome variable).** Participants will be required to describe the Cookie Theft Picture from the Boston Diagnostic Aphasia Examination. The picture will be displayed for 3 minutes, and participants will be instructed to describe what is happening in the picture and to try to talk in complete sentences using a pace they find most comfortable. Picture description will be Used to assess change in discourse abilities, as measured by the change in the total Content Units (CU) and syllable per CU produced by the participants during connected speech.

9. We will also administer the General Health Questionnaire (GHQ, Goldberg & Hillier, 1979) and quality of life (measured by Stroke and Aphasia Quality of Life Scale (SAQOL, Hilari et al., 2003). Both of these questionnaires are secondary outcome variables.

**Procedure for MRI (Visit 4)**

For all participants who consent to MRI and have no contraindication. Participants will receive structural MRI scanning to determine site of lesion, Resting State Functional Connectivity fMRI, and Diffusion Tensor Imaging.

**Procedures for Treatment (Visit 5-19)**

The following procedures will be performed:

1. Measure the electrode location on the scalp (Right cerebellum: 1 cm below the inion and 4 cm lateral)

2. Soak the 2 sponge electrodes in saline solution and place inside rubber electrode holders.

3. Place the cathode electrode under the designated area on the right cerebellum

located during the electrode positioning process (marked with a star) and reference electrode anode on the right deltoid muscle.

4. Connect the electrode cables to the Soterix tDCS device.

5. Start the tDCS device and enter the code. Soterix clinical trial tDCS device includes a software for true operator blinding where the PI can have preset codes for tDCS and sham trials including the dosage and the clinician will enter the codes and the device will present either tDCS or sham depending on the code that was assigned.

6. The Speech Language Pathologist will start the Semantic Feature Analysis Treatment (SFA). Participants will receive SFA treatment for 60 minutes and tDCS for the first 25 minutes. SFA treatment employed in study will include 50 items and their relevant features from eight semantic categories. Items included in each participant's treatment list will be determined based on performance on a picture-naming task. The naming task will consist of 200 items across eight semantic categories (food (including fruits and vegetables), animals, transportation, clothing, music, sports, furniture, toys). The naming task will be administered once. To qualify for treatment, an item must be named incorrectly. To avoid effects of repeated exposure, items included on the naming task will be constrained such that they do not occur in the primary outcome variable (PNT). Therapy tasks will be administered through a computer with clinician assistance using Microsoft Powerpoint. Participants will be trained on 7-12 items per session depending on each participant’s aphasia severity. The treatment protocol will be adapted from Doyle, Dickey and colleagues (Gravier et al., 2018; Evans et al., 2021). The treatment will proceed according to a series of steps including naming aloud the target picture, generating semantic features, naming aloud the target picture again, and generating a sentence using the target word. Participants will be asked to generate semantic features for each target picture in five categories: group [superordinate category], function [use/action], description [physical properties], context [location], and other/personal [association]. A three-level cueing hierarchy will be used to elicit features, consisting of general prompt (e.g., “How would you describe this?”), followed by a relevant directed question (e.g., “What does this feel like?”) and a binary forced-choice question (e.g., “Is this item smooth or rough?”). Only verbal responses will be accepted during treatment.

7. Record any Adverse Events (AE) experienced during the treatment session or since the last visit on the AE log.

8. Assess and record the participant's comfort rating using the Wong-Baker FACES Pain Rating (Wong & Baker, 1988) Scale following treatment completion.

Procedures for Neurological Examination (Will be administered on visit 1, 2, or visit 3)

All participants will be monitored closely for safety and neurological functioning during the duration of the study. In addition to the comfort ratings recorded daily, a neurological examination will be administered during Visit 1, 2, or 3. If there is any change noted in the neurological status by the clinician during the duration of the study, then Dr. Hillis will be notified, and the neurological examination will be repeated.

*b. Study duration and number of study visits required of research participants.*

Participants will undergo screening, language assessments, and MRI (visits 1-3). Eligible participants will receive treatment for 3-5 weeks (3-5 sessions in a week: total 15 sessions). Study duration will be approximately 7-8 months and the number of visits for each participant will be 23.

*c. Blinding, including justification for blinding or not blinding the trial, if applicable.*

Randomization and Blinding

All participants, the members of the study team who administered the assessments and treatments as well as the study biostatistician performing the statistical analyses will be blinded. Participants will be randomized 1:1 (cathodal tDCS or sham tDCS), stratified by site (JHH vs Howard County), aphasia type (fluent vs. non-fluent, classified using WAB-R), and severity (classified using WAB-R Aphasia Quotient in 4 categories (very severe aphasia: 0–25, severe aphasia: 26–50, moderate aphasia: 51–75, and mild aphasia: 76-93.8). Covariate-adaptive randomization method developed by Pocock and Simon, 1975 will be implemented in REDCap. This method ensures balance on important baseline covariates for each treatment arms by calculating the difference in these covariates (site, aphasia type and severity) each time a participant needs be randomized and then randomizes with high probability (80%) to the arm that corrects the imbalance on covariates. The SLP will enter the baseline and eligibility information of a participant prior to enrollment on REDCap. If the participant’s eligibility is confirmed, then the algorithm implemented in REDCap will evaluate the treatment arm distribution in participants already randomized and then generate treatment allocation group (sham or tDCS) based on the randomization scheme. Each participant will receive a unique six-digit codes (provided by manufacturer of the tDCS stimulator), which will instruct the stimulator to deliver either active stimulation or placebo (sham). These codes will be entered into REDCap prior to starting the study. The study coordinator will enter the codes in REDCap.

*d. Justification of why participants will not receive routine care or will have current therapy stopped*

Participation in this study will not disrupt any current care or therapy.

*e. Justification for inclusion of a placebo or non-treatment group*

Both groups will receive semantic feature analysis treatment, a commonly used treatment for naming deficits in aphasia. It is currently unknown whether or not cerebellar tDCS augments the effect of semantic feature analysis in the chronic phase after stroke. Therefore, a sham group is justified.

*f. Definition of treatment failure or participant removal criteria*

Participants will be removed from the study if they are unable to comply with task instructions or tolerate the tDCS procedure.

*g. Description of what happens to participants receiving therapy when study ends or if a participant’s participation in the study ends prematurely*

When the study ends, participants will continue to receive management with Dr. Hillis or their own neurologist as usual (generally follow-up visits every about 6). If a patient’s participation in the study ends prematurely s/he will still receive care as before. In sum, termination of the study or termination of participation in it will not affect regular therapy he or she may be receiving.

**5. Inclusion/Exclusion Criteria**

Participants must satisfy the following inclusion criteria to be considered eligible for entry into this study:

Inclusion Criteria

Chronic ischemic or hemorrhagic left hemisphere stroke

Fluent speaker of English by self-report

Age 18 or older

6 months post onset of stroke

Diagnosis of aphasia and naming impairment using the Western Aphasia Battery-Revised

Exclusion Criteria

Lesion in the right cerebellum

Previous neurological disorder (other than stroke) affecting the brain, or any other neurodegenerative disorder or psychiatric disorder

Seizures during the previous 6 months

Uncorrected visual loss or hearing loss by self-report

Use of medications that lower the seizure threshold (e.g., methylphenidate)

Use of NMDA antagonists (e.g., memantine)

>80% correct response on the Philadelphia Naming Testing at baseline

History of brain surgery or any metal in the head

Severely impaired auditory comprehension (lower than 2 on the Comprehension subscore on the WAB-R)

Severely limited verbal output (lower than 2 on the Spontaneous Speech rating scale on the WAB-R)

Individuals with severe claustrophobia, cardiac pacemakers or ferromagnetic implants, and pregnant women will be excluded from the MRI portion of the study.

**6. Drugs/Substances/Devices**

1. *The rationale for choosing the drug and dose or for choosing the device to be used.*

tDCS has been established as a valid and reliable tool for at least temporarily affecting brain and behavior with minimal risks. Stimulation will be delivered by a battery-driven constant current stimulator (Soterix device, or a comparable model). The stimulator is not connected to a mainline power source and cannot produce in excess of 4mA of current. We will use non-metallic, conductive rubber electrodes covered by saline-soaked sponges to minimize the potential for chemical reactions at the interface of the scalp or skin and the electrodes.

1. *Justification and safety information if FDA approved drugs will be administered for non-FDA approved indications or if doses or routes of administration or participant populations are changed. N/A*
2. *Justification and safety information if non-FDA approved drugs without an IND will be administered. N/A*

**7. Study Statistics**

1. *Primary outcome variable*

The primary outcome will be defined as the change in accuracy of naming measured by the Philadelphia Naming Test, one week after the end of semantic feature analysis (SFA)

1. *Secondary outcome variables*

In addition to the primary outcome, several secondary analyses will be conducted. (1) Trained Picture Naming. We will assess if tDCS has an effect on naming items trained during treatment (trained picture naming). We will assess change in trained picture naming before treatment to within 1 week after the end of treatment. Follow up testing will be done at one month, three months, and six months after the completion of the treatment. (2) Discourse. We will assess change in discourse abilities, as measured by the change in the total Content Units (CU) and syllable per CU produced by the participants during connected speech. Participants will be required to describe the Cookie Theft Picture from the Boston Diagnostic Aphasia Examination. (3) Functional Communication Skills. We will also measure changes in everyday functional communication skills assessed with the Communication Activities of Daily Living, third edition (CADL-3). (4) Finally, we will administer 3 tests from the Research Outcome Measurement in Aphasia-Core Outcome Set (ROMA-COS) at baseline, 1-week post treatment, and at 1-month, 3-month, and 6-month follow-up time points. The WAB-R will be administered as a part of the baseline testing. We will also assess changes in emotional well-being (measured by General Health Questionnaire (GHQ)-12 and quality of life (measured by Stroke and Aphasia Quality of Life Scale (SAQOL-39).

1. *Statistical plan including sample size justification and interim data analysis*

Statistical Analysis

Aim 1: Determine whether cathodal tDCS to the right cerebellum combined with evidenced-based anomia treatment improves picture naming.

Hypothesis1a: 15 sessions of cathodal tDCS over the right cerebellum + SFA treatment will be associated with greater improvement in naming untrained pictures (as measured by the change in PNT),1-week post treatment, compared to sham + SFA.

Statistical analysis**:** The analyses will follow the Intention-to-treat (iTT) principle *where participants are analyzed based on the group to which they are randomized regardless of early termination, missing data or errors in randomization detected post hoc.* The primary outcome variable will be change in accuracy of naming untrained items as measured by the PNT within 1 week after semantic feature analysis ends.

The primary hypothesis is H_0_: mu_1_ = mu_2_ versus H_A_: mu_1_ $\neq$ mu_2_, where mu_1_ is the mean change in accuracy of naming untrained items between baseline and 1-week post- semantic feature analysis in the tDCS group and mu_2_ is the mean change in accuracy of naming untrained items between baseline and 1 week post semantic feature analysis in the sham group. Average Treatment Effect (ATE) will be estimated using linear regression model with change in accuracy of naming untrained items at 1 week as the dependent variable and group assignment (real tDCS versus sham) as the independent variable. ATE is estimated by the coefficient for the group assignment.

As a secondary analysis, we will consider non-parametric mixed models for analyses of functional response over time. In particular, let Y_ijk_ = u_ik_ + fk(j) + e_ij_ where Y_ij_ is the the outcome for subject i on occasion j (0, 1, 3, 6) within treatment arm k. (Thus, both i and k are necessary to identify a subject). No covariates are necessary because of the randomization (though see Aim 2). f_k_(j) is a functional model we will estimate using quadratic regression splines with knot points at each of the time points. Given there are so few time points, we will not penalize the spline fit. A non-parametric estimate of a treatment effect is given by f_2_ – f_1,_ which can show time-specific treatment effects when evaluated at specific points j. This will also demonstrate the rate (when and if) at which TCDS effects ebb. An overall effect can be estimated by simply taking the integral of f_2_ – f_1_ (i.e. the functional averaged effect over time). A null hypothesis of zero represents no time averaged effect of the treatment. Given that we will use regression splines, every estimator reduces to standard contrasts of regression parameters, and thus can be implemented in any statistical software package.

Hypothesis 1b: For secondary outcomes, we hypothesize that cathodal cerebellar tDCS results in greater improvement in trained picture naming, greater improvement in discourse (as measured by total content units (CU) and syllable per CU in picture description) and greater improvement in functional communication skills (as measured by change in Communication Activities of Daily Living–CADL-3) compared to sham.

Statistical Analysis: We will evaluate the effect of tDCS on naming trained items, discourse and functional communication skills. In addition, we will also evaluate the effect of tDCS on WAB-R Aphasia Quotient, SAQOL, and GHQ.The analyses will follow similar approach to Aim 1a.

Aim 2: To identify whether neural (functional and structural) biomarkers and linguistic characteristics can predict response to cerebellar stimulation and SFA treatment.

Hypothesis2: Baseline resting state functional connectivity, structural connectivity, lesion volume and site, and overall aphasia severity and naming severity will predict response to cerebellar tDCS treatment.

Statistical analysis for Aim 2**:** This analysis considers moderation of treatment effects by pre-treatment baseline characteristics. The pre-treatment baseline characteristics include the following: Imaging: Structural (lesion volume, site, FA, MD), Functional (Fisher transformed connectivity values (*z* scores); Linguistic: (Aphasia Severity score as assessed by WAB-R, Naming severity score assessed by PNT). As in Hypothesis 1, we will consider both a conservative approach, using standard contrasts and median splits on the moderating variables as well as a mixed model functional approach. That is, we will split moderating measures, such as the rs-fMRI Fisher Z-transformed measures of connectivity at the median values, then will test the hypothesis that the tDCS response difference varies between the below median values and the above. A standard t-test contrast approach will be adjusted for multiplicity using Bonferroni corrections given that we will separately analyze potential treatment moderators. We will focus on most proximal post treatment outcomes as our primary test, as the power for detecting moderation will likely be the greatest where the treatment effect is strongest. Our second approach will use a fully model based approach that will be able to incorporate multiple moderators simultaneously. This will not require dichotomizing the moderating variables and it will model the relationship non-parametrically. We build on the functional mixed model approach outlined before. Consider building on our model as Y_ijk_ = u_ik_ + x_i_ B + f_k_(j, x_i_) + e_ij_ where here x_i_ is a vector of pretreatment characteristics of interest, such as a collection of lesion volumes or inter-regional Fisher Z-transformed connectivity, i is subject, k is treatment status and j is sampling occasion (time), f_k_(j, x_i_) is the non-parametric interaction effect between treatment status and the potential interaction moderating variables. Note that the function f_k_(j, x_i_) is now a function of both time and baseline participant characteristics. Therefore, we will follow our testing strategy as before. However, of interest is whether f_2_ – f_1_ is flat with respect to dependence on xi. Specifically, consider the simpler model where Y_ijk_ = u_ik_ + x_i_ B + q_k_(j) + e_ij_ where note that the treatment effect, represented by q_k_(j) in the simpler model, does not depend on the moderating variables. Testing between the more complex and simpler models is a test of whether the treatment effect is influenced by the moderating variables. Inference will be performed via the bootstrap. Of note, flatness f_2_ – f_1_ with respect to the moderating variables is a strong null. We will also consider a weaker null under linear separability: f_2_(j, x_i_) = g_k_(j) + h_k_(x_i_). Here the non-parametric treatment function allows for a non-linear effect of the potential moderating variables where they do not have any actual interaction with the treatment effect. While this overall approach is complex, it offers a complete picture of treatment response over time and the impact that moderating variables have. In addition, the model allows us to fit much more complex potential relationships between the impact of treatment and the moderating variables and allows us to consider the moderating variables simultaneously. To summarize the approaches, we will proceed in this order

1. T-test comparing the treatment effect across median splits of the moderating variables performed separately, one at a time.
2. A non-parametric modeling approach using spline based linear models testing whether the potential moderating variables interact with the treatment effect over time. We will consider two variations of this approach:
   1. One that assumes linearity
   2. One that assumes non-parametric functions

Sample Size Determination

We used the data from the PI’s K99 study (crossover trial) to estimate the variability of untrained naming score. 52 participants (26 per group) will give us 80% statistical power to detect 0.7 SD difference in change in accuracy of naming untrained items at 1-week post treatment between the study arms. This was done using Wald test for group assignment coefficient in linear regression at 0.1 level of statistical significance. The effect size (0.7SD) is a bit conservative compared to the difference observed on group comparison for 21 participants (10 in tDCS and 11 in sham) in the K99 data, when the tDCS was administered in Phase 1. We propose to enroll 60 participants to account for 10% attrition.

Missing Data

*The analyses will follow the Intention-to-treat (iTT) principle where participants are analyzed based on the group to which they are randomized regardless of early termination, missing data or errors in randomization detected post hoc. To minimize possible biases, analyses will be by intention to treat; any missing data will be addressed with the technique of multiple imputation (Ball et al., 2002; Rubin, 2009), generally recognized as best for handling missing data (Little et al., 2012).*

1. *Early stopping rules. N/A*

**8. Risks**

1. *Medical risks, listing all procedures, their major and minor risks and expected frequency.*

tDCS

tDCS provides a non-invasive method to stimulate the cortex and cerebellum and modulate cortical/cerebellar activity via continuous, weak polarizing electrical current. This study will use the Soterix Medical 1X1 Clinical Trials system to administer tDCS. The Soterix transcranial Direct Current Stimulator Clinical Trials (1x1-CT) system is the most advanced and customizable stimulation for true double-blind control trials. It is powered by 4 9 V battery with an output of 1-2.5 milliamperes (mA). Anodal tDCS (A-tDCS) results in increase in cortical excitability. Cathodal tDCS results in decrease in cortical excitability. To date, no serious adverse effects of tDCS have been reported in the literature as long as safety guidelines are followed Lefaucheur et al., 2017; Fregni et al., 2021). A recent review updated and consolidated the evidence on the safety of tDCS (Bikson et al., 2016). This review shows that the use of conventional tDCS protocols in human trials (≤40 min, ≤4 mA) has not produced any reports of a serious adverse effect or irreversible injury across over 33,200 sessions and 1000 subjects with repeated sessions. This includes a wide variety of subjects, including participants with stroke. Very minor side effects such as itching, tingling, burning have been reported, as well as temporary headache, sleepiness, dizziness. However, they were generally indistinguishable from those reported by participants receiving sham stimulation. The current study will only administer 2 mA for 25 minutes per treatment session. It is important to note that tDCS does not cause significant heating effects under the electrodes, alter the blood-brain barrier, or induce edema.

Our recent study in chronic post stroke aphasia (20 min, 2mA) in 24 participants did not produce any negative effects associated with tDCS administration beyond mild itching/tingling at the beginning of the treatment session (Sebastian et al., 2020). A recent large crossover trial in 36 participants with Primary Progressive Aphasia (20 min, 2mA) reported no episodes of intolerability and no serious adverse effects (Tsapkini et al., 2018). On the Wong-Baker FACES Pain Rating Scale, the mean pain rating for tDCS was 2.21 (standard deviation 2.48, range 0–10) and the mean rating for sham was 2.14 (standard deviation 2.13, range 0–10). Another large, randomized control trial in 74 participants with aphasia reported 8 mild, non-serious adverse events and there were no statistically significant differences between treatment groups for number of adverse events (Fridriksson et al., 2018). 2 participants (6%) in the active tDCS group experienced transient scalp redness/irritation (erythema) compared with none in the sham tDCS group. On the Wong-Baker FACES Pain Rating Scale, most often individuals reported no hurt: 94% (n = 476) in the active tDCS group vs 86% (n = 511) in the sham group. The highest pain rating reported was 3 (indicating “hurts even more”), which was reported 4 times by 2 individuals (3%), both in the sham group. Taken together, all available research suggests that prolonged application should not pose a risk of brain damage when applied according to safety guidelines.

MRI

Participants may undergo MRI scanning in the present study. The effects of undergoing MR scanning have been extensively studied and there are no risks associated with an MR exam. The patient may, however, be bothered by feelings of confinement (claustrophobia), and by the noise made by the magnet during the procedure. They will be asked to wear earplugs or earphones while in the magnet.

1. *Steps taken to minimize the risks.*

Participants will be carefully screened over the phone prior to being scheduled, to assure that they meet study criteria. tDCS stimulation will be ramped up over the first 15 seconds of stimulation in order to eliminate the sensation of tingling that can occur under the electrodes during the initial moments of tDCS application. The participant may stop testing or the intervention any time. There will be emergency personnel and equipment on hand for your safety.

1. *Plan for reporting unanticipated problems or study deviations.*

Adverse events will be monitored during the entire visit by the study team. The families will be given telephone numbers of study team as well. The study physician (Dr. Argye Hillis) and the DSMB will be notified immediately if any adverse events are reported. The DSMB will determine if the adverse event is a serious adverse event. Adverse events will be monitored until they are resolved or clearly determined to be due to a subject’s stable or chronic condition or intercurrent illness. In the case of any unexpected adverse events involving risks to participants or others that are related/possibly related to the research, a Protocol Event Report will be prepared by the Study Coordinator, the PI will be informed immediately, and the IRB will be contacted within 10 days as per Johns Hopkins Medicine IRB policy; deaths will be reported within 72 hours. Also, as required by IRB policy, any unexpected adverse device effects, potential breaches of confidentiality, unresolved participant complaints will be promptly reported to the IRB. Any other adverse events that do not require prompt reporting will be summarized and reported to the IRB at the time of continuing review.

Plan for dealing with incidental findings: All MRI scans will be reviewed by Co-I and board-certified neurologist (Dr. Argye Hillis), and any suspicious abnormalities will be referred to a board-certified neuroradiologist. If unexpected abnormalities - incidental findings - are seen (which is unlikely, as every patient will have had a clinical MRI as part of their evaluation for stroke), the patient will be asked permission to contact the primary care physician about the abnormality, and will be offered a timely appointment with a neurologist (Argye E. Hillis, MD) if appropriate.

1. *Legal risks such as the risks that would be associated with breach of confidentiality.*

Participation in this study should not put participants in any legal risk, even in the case of a breach of confidentiality. We will undertake every effort to keep the information in the study confidential. Participants will be assigned a code number for the scans in order to keep the information confidential. Consent forms and source documents will be maintained at the PI lab in locked cabinet. All digital data will be stored on a password-protected and encrypted format in a manner that is Johns Hopkins IRB compliant.

This will include the Clinical Research Management System (CRMS), Research Electronic Data Capture (REDCap), and Johns Hopkins Microsoft One Drive. All are web-based applications designed to organize and streamline clinical research management. CRMS is integrated with Epic, Hopkins enterprise EMR, as well as Johns Hopkins IRB. This integration improves communication among study team members, store subject enrollment information in a secure location, assists with recruitment, and allows research results to be promptly incorporated into the EMR. Everybody involved in the study will have completed the appropriate HIPAA training and are fully aware of confidentiality issues. No names will be included in any publications resulting from this work.

1. *Financial risks to the participants.*

No financial risk is involved. Only participants who are interested in trying word retrieval therapy with tDCS and can be in Baltimore for the therapy as well as the follow-up sessions will participate in the study.

**9. Benefits**

1. *Description of the probable benefits for the participant and for society.*

We cannot ensure that this research will provide any direct, sustainable benefit to the participants. It is possible that most participants will benefit from the present therapeutic intervention. Participants may or may not learn strategies to facilitate word retrieval and this knowledge may or may not generalize to other items or functions. Completion of this project will result in better understanding whether and how tDCS coupled with behavioral therapy may help individuals with post stroke aphasia.

**10. Payment and Remuneration**

1. *Detail compensation for participants including possible total compensation, proposed bonus, and any proposed reductions or penalties for not completing the protocol.*

Participants will not be paid to participate in the study. There is no penalty for not completing a tDCS session. Participants will be reimbursed $50 for travel and parking for each therapy session and evaluation session if they provide their own transportation. The study will provide transportation for patients who do not have transportation (via a sedan service used frequently by Johns Hopkins)

**11. Costs**

1. *Detail costs of study procedure(s) or drug (s) or substance(s) to participants and identify who will pay for them.*

There is no cost to the participants for participating in the study.

**12. Transfer of Materials**

N/A

**12. References**

Baker JM, Rorden C, Fridriksson J. Using transcranial direct-current stimulation to treat stroke patients with aphasia. Stroke. 2010;41(6):1229-36

Ball K, Berch DB, Helmers KF, Jobe JB, Leveck MD, Marsiske M, Morris JN, Rebok GW, Smith DM, Tennstedt SL, Unverzagt FW. Effects of cognitive training interventions with older adults: a randomized controlled trial. Jama. 2002 Nov 13;288(18):2271-81.

Bhogal SK, Teasell R, Speechley M. Intensity of aphasia therapy, impact on recovery. Stroke. 2003 Apr 1;34(4):987-93.

Bikson M, Grossman P, Thomas C, Zannou AL, Jiang J, Adnan T, Mourdoukoutas AP, Kronberg G, Truong D, Boggio P, Brunoni AR. Safety of transcranial direct current stimulation: evidence based update 2016. Brain Stim. 2016;9(5):641-61.

Boyle M. Semantic feature analysis treatment for aphasic word retrieval impairments: What’s in a name?. Topics in Stroke Rehab. 2010;17(6):411-22.

Boyle M, Coelho CA. Application of semantic feature analysis as a treatment for aphasic dysnomia. American J Speech-Lang Path. 1995;4(4):94-8.

Breining BL, Lala T, Martínez Cuitiño M, Manes F, Peristeri E, Tsapkini K, Faria AV, Hillis AE. A brief assessment of object semantics in primary progressive aphasia. Aphasiology. 2015 Apr 3;29(4):488-505.

Breining BL, Sebastian R. Neuromodulation in post-stroke aphasia treatment. Current physical medicine and rehabilitation reports. 2020 Jun;8(2):44-56.

Brunoni AR, Nitsche MA, Bolognini N, Bikson M, Wagner T, Merabet L, Edwards DJ, Valero-Cabre A, Rotenberg A, Pascual-Leone A, Ferrucci R. Clinical research with transcranial direct current stimulation (tDCS): challenges and future directions. Brain Stim. 2012;5(3):175-95.

Datta A, Baker JM, Bikson M, Fridriksson J. Individualized model predicts brain current flow during transcranial direct-current stimulation treatment in responsive stroke patient. Brain Stim. 2011;4(3):169-74.

Desmond JE, Fiez JA. Neuroimaging studies of the cerebellum: language, learning and memory. Trends Cog Sci. 1998;2(9):355-62.

de Aguiar V, Paolazzi CL, Miceli G. tDCS in post-stroke aphasia: the role of stimulation parameters, behavioral treatment and patient characteristics. Cortex. 2015 Feb 1;63:296-316.

DeMarco AT, Dvorak E, Lacey E, Stoodley CJ, Turkeltaub PE. An Exploratory Study of Cerebellar Transcranial Direct Current Stimulation in Individuals With Chronic Stroke Aphasia. Cog Behavior Neuro. 2021;34(2):96-106.

De Smet HJ, Paquier P, Verhoeven J, Mariën P. The cerebellum: its role in language and related cognitive and affective functions. Brain Lang. 2013;127(3):334-42.

Dmochowski JP, Datta A, Huang Y, Richardson JD, Bikson M, Fridriksson J, Parra LC. Targeted transcranial direct current stimulation for rehabilitation after stroke. Neuroimage. 2013 Jul 15;75:12-9.

Evans WS, Cavanaugh R, Gravier ML, Autenreith AM, Doyle PJ, Hula WD, Dickey MW. Effects of Semantic Feature Type, Diversity, and Quantity on Semantic Feature Analysis Treatment Outcomes in Aphasia. American J Speech-Lang Path. 2021;30(1S):344-58.

Fregni F, El-Hagrassy MM, Pacheco-Barrios K, Carvalho S, Leite J, Simis M, Brunelin J, Nakamura-Palacios EM, Marangolo P, Venkatasubramanian G, San-Juan D. Evidence-based guidelines and secondary meta-analysis for the use of transcranial direct current stimulation (tDCS) in neurological and psychiatric disorders. International J Neuropsychopharmacology. 2021;24(4):256-313.

Fridriksson J, Richardson JD, Baker JM, Rorden C. Transcranial direct current stimulation improves naming reaction time in fluent aphasia: a double-blind, sham-controlled study. Stroke. 2011;42(3):819-21.

Fridriksson J, Richardson JD, Fillmore P, Cai B. Left hemisphere plasticity and aphasia recovery. Neuroimage. 2012;60(2):854-63.

Fridriksson J, Rorden C, Elm J, Sen S, George MS, Bonilha L. Transcranial direct current stimulation vs sham stimulation to treat aphasia after stroke: a randomized clinical trial. JAMA Neurol. 2018;75(12):1470–6.

Fritsch B, Reis J, Martinowich K, Schambra HM, Ji Y, Cohen LG, Lu B. Direct current stimulation promotes BDNF-dependent synaptic plasticity: potential implications for motor learning. Neuron. 2010 Apr 29;66(2):198-204.

Galea JM, Jayaram G, Ajagbe L, Celnik P. Modulation of cerebellar excitability by polarity-specific noninvasive direct current stimulation. J Neurosci. 2009; 29: 9115-22.

Goldberg DP, Hillier VF. A scaled version of the General Health Questionnaire. Psychological Med. 1979;9(1):139-45.

Goodglass H, Kaplan E, Barresi B. BDAE-3: Boston Diagnostic Aphasia Examination–Third Edition. Philadelphia, PA: Lippincott Williams & Wilkins; 2001.

Gravier ML, Dickey MW, Hula WD, Evans WS, Owens RL, Winans-Mitrik RL, Doyle PJ. What matters in semantic feature analysis: Practice-related predictors of treatment response in aphasia. American J Speech-Lang Path. 2018;27(1S):438-53.

Grimaldi G, Argyropoulos GP, Bastian A, Cortes M, Davis NJ, Edwards DJ, Ferrucci R, Fregni F, Galea JM, Hamada M, Manto M. Cerebellar transcranial direct current stimulation (ctDCS) a novel approach to understanding cerebellar function in health and disease. The Neuroscientist. 2016 Feb;22(1):83-97.

Hamilton RH, Chrysikou EG, Coslett B. Mechanisms of aphasia recovery after stroke and the role of noninvasive brain stimulation. Brain and language. 2011;118(1-2):40-50.

Hartwigsen G, Saur D. Neuroimaging of stroke recovery from aphasia–Insights into plasticity of the human language network. Neuroimage. 2019; 190:14-31.

Helm-Estabrooks N. Cognitive linguistic quick test: CLQT. Psychological Corporation; 2001.

Hillis AE. Treatment of naming disorders: New issues regarding old therapies. Journal of the International Neuropsychological Society. 1998 Nov;4(6):648-60.

Hilari K, Byng S, Lamping DL, Smith SC. Stroke and aphasia quality of life scale-39 (SAQOL-39) evaluation of acceptability, reliability, and validity. Stroke. 2003;34(8):1944-50.

Holland R, Crinion J. Can tDCS enhance treatment of aphasia after stroke?. Aphasiology. 2012 Sep 1;26(9):1169-91.

Holland AL, Wozniak L, Fromm D. CADL-3: Communication Activities of Daily Living. Pro-ed; 2018.

Howard D, Patterson K. The Pyramids and Palm Trees Test: A test of semantic access from words and pictures. Pearson Assessment; 1992.

Kertesz A. Western aphasia battery: Revised. Pearson; 2007.

Kaplan, E., Goodglass, H., & Weintraub, S. Boston naming test-2. Austin TX: Pro-Ed; 2001.

Kiran S, Thompson CK. The role of semantic complexity in treatment of naming deficits. *J Speech, Lang, Hearing Research*. 2003; *46*(3), 608-622.

Lang N, Siebner HR, Ward NS, Lee L, Nitsche MA, Paulus W, Rothwell JC, Lemon RN, Frackowiak RS. How does transcranial DC stimulation of the primary motor cortex alter regional neuronal activity in the human brain?. European Journal of Neuroscience. 2005 Jul;22(2):495-504.

Lefaucheur JP, Antal A, Ayache SS, Benninger DH, Brunelin J, Cogiamanian F, et al. (2017). Evidence-based guidelines on the therapeutic use of transcranial direct current stimulation (tDCS). Clin. Neurophysiol. 128, 56–92.

Little RJ, D'Agostino R, Cohen ML, Dickersin K, Emerson SS, Farrar JT, Frangakis C, Hogan JW, Molenberghs G, Murphy SA, Neaton JD. The prevention and treatment of missing data in clinical trials. NJEM. 2012;367(14):1355-60.

Maddy KM, Capilouto GJ, McComas KL. The effectiveness of semantic feature analysis: An evidence-based systematic review. Annals of Phys Rehab Med. 2014;57(4):254-67.

Marangolo P, Fiori V, Caltagirone C, Pisano F, Priori A. Transcranial cerebellar direct current stimulation enhances verb generation but not verb naming in poststroke aphasia. J Cog Neurosci. 2018;30(2):188-99.

Murdoch BE. The cerebellum and language: historical perspective and review. Cortex. 2010;46(7):858-68.

Nitsche MA, Paulus W. Excitability changes induced in the human motor cortex by weak transcranial direct current stimulation. J Physio. 2000;527(Pt 3):633.

Raymer AM, Kohen F, Blonder LX, Douglas E, Sembrat JL, Rothi LJ. Effects of gesture and semantic-phonologic treatments for noun retrieval in aphasia. Brain and Language. 2007;1(103):219-20.

Rioult-Pedotti MS, Friedman D, Donoghue JP. Learning-induced LTP in neocortex. Science. 2000;290(5491):533-536.

Roach A, Schwartz MF, Martin N, Grewal RS, Brecher A. The Philadelphia naming test: scoring and rationale. Clinical Aphasio.1996; 24: 121-33.

Rubin DB. Multiple imputation for nonresponse in surveys. John Wiley & Sons; 2004 Jun 9.

Sebastian R, Saxena S, Tsapkini K, Faria AV, Long C, Wright A, Davis C, Tippett DC, Mourdoukoutas AP, Bikson M, Celnik P. Cerebellar tDCS: a novel approach to augment language treatment post-stroke. Front Hum Neurosci. 2017;10:695

Sebastian R, Kim, J H, Brenowitz R, Tippett, DC, Desmond, JE, Celnik P, Hills AE. Cerebellar neuromodulation improves naming in post-stroke aphasia. Brian Comm. 2020;2(2):fcaa179

Strand EA, Duffy JR, Clark HM, Josephs K. The apraxia of speech rating scale: A tool for diagnosis and description of apraxia of speech. J Commun Disord. 2014; 51:43–50.

Tsapkini K, Webster KT, Ficek BN, Desmond JE, Onyike CU, Rapp B, Frangakis CE, Hillis AE. Electrical brain stimulation in different variants of primary progressive aphasia: A randomized clinical trial. Alzheimer's & Dementia: Translational Research & Clinical Interventions. 2018 Jan 1;4:461-72.

Turkeltaub PE, Swears MK, D’Mello AM, Stoodley CJ. Cerebellar tDCS as a novel treatment for aphasia? Evidence from behavioral and resting-state functional connectivity data in healthy adults. Restorative Neuro Neurosci. 2016;34(4):491-505.

Wallace SJ, Worrall L, Rose T, Le Dorze G, Kirke E, Kolomeitz D. Report from ROMA: an update on the development of a core outcome set for aphasia research. Aphasiology. 2018;32(sup1):241-2.

Weiss SA, Bikson M. Open questions on the mechanisms of neuromodulation with applied and endogenous electric fields. Frontiers in human neuroscience. 2014 Apr 17;8:227.

Wisenburn B, Mahoney K. A meta-analysis of word-finding treatments for aphasia. Aphasiology. 2009 Oct 15;23(11):1338-52.

Wong, D.L., & Baker, C. (1988). Pain in children: Comparison of assessment scales. Pediatric Nursing, 14(1), 9-17.
