# Supplementary material for: Protocol for Cerebellar Stimulation for Aphasia Rehabilitation (CeSAR): A randomized, double-blind, sham-controlled trial
Source: PLoS One. 2024 Aug 26;19(8):e0298991. doi: 10.1371/journal.pone.0298991 (PMC11346736; doi:10.1371/journal.pone.0298991)
Supplement: S3 File — (PDF) [file pone.0298991.s003.pdf]

If appropriate for this study, a scanned copy of the signed consent form should be uploaded to the participant's Epic/EMR record.

Patient I.D. plate

RESEARCH PARTICIPANT INFORMED CONSENT AND PRIVACY AUTHORIZATION FORM

Protocol Title: **Cerebellar Stimulation for Aphasia Rehabilitation**

Application No: IRB00300301

Sponsor: National Institute on Deafness and Other Communication Disorders

Principal Investigator: Rajani Sebastian, PhD, CCC-SLP
Department of Physical Medicine and Rehabilitation
Johns Hopkins School of Medicine
600 N. Wolfe Street, Phipps-183
Baltimore, MD 21287
Tel: (+) 1-410-502-5012
Fax: (+) 1-410-502-2419

You are being asked to join a research study. Participation in this study is voluntary. Even if you decide to join now, you can change your mind later.

The person being asked to be in this research study may not be able to give consent to be in this study. You are therefore being asked to give permission for this person to be in the study as his/her decision maker.

1. Research Summary (Key Information):

The information in this section is intended to be an introduction to the study only. Complete details of the study are listed in the sections below. If you are considering participation in the study, the entire document should be discussed with you before you make your final decision. You can ask questions about the study now and at any time in the future.

This research is being done to help us understand whether the application of transcranial direct current stimulation (tDCS) along with naming therapy may improve naming in people with post-stroke aphasia (trouble understanding and talking).

- tDCS is the application of a very weak electrical current on the surface of the scalp. Most individuals do not find the tDCS procedure uncomfortable, and there are no known long-term risks of tDCS. When the tDCS current goes through the electrodes, you may feel an itching or tingling sensation under the electrodes or see slight light flashes, or you may not feel anything at all.
- Naming therapy is commonly used to help aphasia patients find words and language through the use of cues and pictures.

Participants will be randomized to receive either active tDCS or "sham" tDCS condition along with naming therapy. For the "sham" tDCS condition, the electrodes will be placed on your skin, but electrical current will be administered only for the first few seconds. Each participant will take part in 15 sessions of naming therapy combined either active tDCS or sham tDCS. At baseline, each participant will receive language and cognitive evaluations, and undergo brain-imaging assessments (using Magnetic Resonance Imaging or MRI scans). The same language and cognitive evaluation will be carried out at the end of 15 treatment sessions of either tDCS or sham plus naming therapy.

Participants will undergo a screening period, which will include language assessments and MRI (this can be done over 1-4 visits). Eligible participants will receive tDCS or sham tDCS for 3-5 weeks (3-5 sessions in a week: total 15 sessions). The study duration will be approximately 7-8 months and the number of visits for each participant will be 23.

There are risks to the study procedures such as tDCS and the MRI scan that are described later in this document. There may or may not be a benefit to you from participating in this study.

2. Why is this research being done?

This research is being done to help us understand whether and how the application of transcranial direct current stimulation (tDCS) along with naming therapy, may affect recovery of naming problems in people with aphasia (trouble understanding and talking) caused by stroke.

Our aim is to find out whether tDCS in combination with naming therapy will improve how people with aphasia perform on tasks such as recalling words. Another goal is to identify stroke patients who are most likely to benefit from tDCS. We will combine language assessment and brain imaging measures to identify the characteristics of stroke patients that predict the greatest benefit from tDCS.

Are there any investigational drugs/devices/procedures?

This research study will utilize Soterix Medical device to deliver tDCS. The use of "Soterix Medical tDCS device" in this research study is investigational. The word "investigational" means that "Soterix Medical tDCS device" is not approved for marketing by the Food and Drug Administration (FDA).

Who can join this study?

People 18 years and older diagnosed with aphasia caused by stroke may join.

How many people will be in this study?

About 60 people are expected to take part.

3. What will happen if you join this study?

If you agree to be in this study, we will ask you to do the following things:

Language and Cognitive Evaluation

You will be asked to first complete general medical, tDCS and Magnetic Resonance Imaging (MRI) screening questionnaires. You will also complete a neurological examination. After the completion of the screening questionnaires, you will have an evaluation of your language and cognitive skills. The screening procedures and neurological examination will take 30 minutes and the language and cognitive evaluations will take about 3-4 hours. The evaluations can be completed over multiple sessions if necessary.

Naming Therapy

If naming therapy is appropriate for you based on the results from these assessments, you will then begin naming therapy. You will engage in naming therapy for 15 sessions over the course of 3-5 weeks, depending on your availability. The therapy session will last 1 hour. Naming therapy will use meanings and cues of certain words to help participants recall (find) words.

Transcranial Direct Current Stimulation (tDCS)

tDCS will be applied while you are engaged in naming therapy. Participants will receive active tDCS or "sham" tDCS by random assignment, like flipping a coin. One half of the participants (1 in 2) will receive "sham" tDCS and one half (1 in 2) will receive active tDCS.

For the "sham" tDCS, the electrodes will be placed on your skin, but electrical current will be administered only for the first few seconds. All participants, whether they receive active tDCS or "sham" tDCS, will receive the same naming therapy.

We will place small electrodes inserted into a sponge that have been soaked with saline on your scalp: one electrode will be placed on the back side of your head and the other electrode will be placed on the right shoulder. For the "sham" tDCS, the electrodes will be placed on your scalp, but electrical current will be administered only for the first few seconds. Once the electrodes are in place, you will start naming therapy with a Speech Language Pathologist and a small electrical current will be passed between the electrodes. tDCS will last for 25 minutes and during this time you will be engaged in naming therapy. After the completion of stimulation, naming therapy will continue for another 35 minutes.

Neither you nor Speech Language Pathologist will know whether you receive active tDCS and sham tDCS. In the case of an emergency, the study doctor can quickly find out which intervention you are assigned to receive.

Follow-Up Visits

You will be asked to undergo 4 additional follow-up visits (Visits 20-23), at one week, 1 month, 3 months and 6 months post intervention, which will include language and thinking assessments. In order to minimize the need for research-only in-person visits, the follow up visits may be done remotely.

Video/Audio recordings

As part of this research, we will create and use video and audio recording of the language evaluations/intervention sessions to help answer the research questions. Study staff will watch and listen to the recordings to score and analyze your language evaluations and response during the intervention sessions. Any video and audio recording will not be used for advertising or non- study related purposes.

You should know that:

- You may request that the audio and video recording be stopped at any time.
- If you agree to allow video and audio recordings and then change your mind, you may ask us to destroy that imaging/recording. If the imaging/recording has had all identifiers removed, we may not be able to do this.
- We will only use these recordings for the purposes of this research.

Please indicate your decision below by checking the appropriate statement:

YES ☐ I **agree** to allow the Principal Investigator and Johns Hopkins study team members to make and use photographs/video recordings/audio recordings of me (or the participant I represent) for the purpose of this study.

NO ☐ I **do not agree** to allow the Principal Investigator and Johns Hopkins study team members to make and use photographs/video recordings/audio recordings of me (or the participant I represent) for the purpose of this study.

____ _______________

Participant Signature Date
(or Parent/Legally Authorized Representative Signature, if applicable)

MRI

As part of your participation in this research study, you may be asked to have a Magnetic Resonance Imaging (MRI) exam before the start of naming therapy. You may decline to have the MRI exam and still participate in the study. Not all participants will be eligible to take part in the MRI part of this study.

Magnetic Resonance Imaging (MRI) scans create images of the body using a magnet and radio waves. There is no radiation involved in an MRI exam. The MRI exam(s) in this study will take about 60 minutes.

You may not take part in this part of the study if you have any metal or device in your body which is not compatible with MRI. Examples include certain pacemakers, defibrillators, aneurysm clips, or certain other implanted electronic or metallic devices, shrapnel, or other metal.

If you have a history of metal in your head or eyes, you cannot take part in the MRI part of this study.

The MRI machine periodically makes loud banging noises. We will provide earplugs or headphones for you to wear during the MRI exam.

Incidental Findings

As part of this research study, you will undergo an imaging procedure. A qualified professional will review your research imaging. This research imaging will not include the full diagnostic information that you would get if your primary doctor referred you for imaging.

There is a possibility that while reviewing your imaging we may see an unexpected abnormality. This is called an "incidental finding."

We will let you know if we see such an incidental finding. Depending on the type of incidental finding, we may contact you by mail, email, or phone. In the case of a potential serious emergency, someone may go to your home.

A qualified person (usually a member of the research team) will talk to you if there is an incidental finding. <u>You do not have an option to decline information about an incidental finding from an imaging procedure.</u>

If you want, we will give information about this incidental finding to your primary doctor or we will refer you to an appropriate doctor for further evaluation.

What could happen if there is an incidental finding?

- An incidental finding may cause you to feel anxious.
- Since a report of the incidental finding will be part of your medical record, it will be available to those accessing your medical record for your clinical care and may affect your current or future life or health insurance coverage. This risk will vary depending on the type of insurance plan involved.

The costs for any care that may come from the incidental finding, such as the need to see a doctor to diagnose or treat an incidental finding, will not be paid for by this research study. These costs would be your or your insurance company's responsibility.

<u>Will research test results be shared with you?</u>
It is uncertain if the research tests will produce results that would be relevant for your clinical care, so we will not share these results with you.

<u>How long will you be in the study?</u>
You will be in this study for 7-8 months including follow-up evaluations, depending on your availability.

4. What happens to data that are collected in the study?

If you join this study, your data will be used to answer the research question and publish the findings of this study. You will not own your research data. If researchers use your data to create a new product or idea, including those that may have commercial value, you will not benefit financially from those efforts.

Johns Hopkins researchers and their collaborators may use the data collected in this study for future research purposes and may share some of the data with others.

Sharing data is part of research and may increase what we can learn from this study. Often, data sharing is required as a condition of funding or for publishing study results. It also is needed to allow other researchers to validate study findings and to come up with new ideas. Your data may be shared with researchers at Johns Hopkins and other institutions, for-profit companies, sponsors, government agencies, and other research partners. Your data may also be put in government or other databases/repositories.

Because science constantly advances, we do not yet know what future use of research data may include. This future research may be unrelated to the current study and may include outside collaborators.

We (Johns Hopkins) will do our best to protect and maintain your data in a safe way. One of the ways we protect data is by limiting the uses of the information and the type of information that is shared, especially your personal information. This may occur through data sharing agreements and review by oversight groups within Johns Hopkins.

If data are used or shared with types of information that may be likely to identify you, such as your name, address or medical record number, further institutional review and approval would be required. In these cases, Johns Hopkins will review whether additional consent from you is required.

Generally, if your data are used/shared without any personal identifiers or with information that is less likely to identify you (such as the date of a procedure), further review and approval is not needed.

Data sharing could change over time, and may continue after the study ends.

The use and sharing of your data is required for participation in this research study. If you are not comfortable with the use and sharing of your data in future research without further consent, you should not participate in this study.

5. What are the risks or discomforts of the study?

tDCS

tDCS has been used in humans and animals for many years. In recent studies that involved several hundred people, very minor side effects such as itching, tingling, or burning have been reported under the electrode that went away when the current was stopped. However, they were generally indistinguishable from those reported by participants receiving sham stimulation.

There is no danger of heat to the brain during tDCS. However, there is a slight risk of an electric burn. One patient without adequate protection had a small burn on the ear, which healed in several days. To eliminate this risk, we will use insulated carbon electrodes. If you develop any problem during any of the experiments, we will stop the stimulation immediately. Any effect on brain function will be brief.

Language and Cognitive Evaluations and Therapy

You may get tired or bored when we are asking you questions or when you are completing speech and language evaluation and therapy. If you feel tired or frustrated, you may take a break. You do not have to answer any question you do not want to answer.

MRI

While no significant risks have been found from the use of MRI scans, you may be bothered by the noise made by the MRI scanner and by feelings of being closed in (claustrophobia).

Identifiable private information

There is the risk that information about you may become known to people outside this study.

Unknown risk

There may be side effects and discomforts that are not yet known.

6. Are there benefits to being in the study?

You may or may not benefit from being in this study. If you take part in this study, you may help others in the future.

7. What are your options if you do not want to be in the study?

You do not have to join this study. If you decide not to join this study, other options are available. You do not have to join this study to get treatment. Other treatments include referral to a speech-language pathologist for treatment of language deficits. If you do not join, your care at Johns Hopkins will not be affected.

8. Will it cost you anything to be in this study?

You will receive a separate Insurance and Research Participant Financial Responsibility Information Sheet (Sheet). This Sheet will give you the following information:

- The procedures, tests, drugs or devices that are part of this research and that will be paid for by the study (no cost to you).
- The procedures, tests, drugs or devices that will be billed to you and/or your health insurer. If you have health insurance, you will be responsible for any co-pays or deductibles not covered by your insurance.

9. Will you be paid if you join this study?

You will be paid $50 per visit to cover your transportation to the hospital and parking, if you provide your own transportation. If we provide transportation, you will not be paid. We will pay you at the first follow-up session (within 1 week after treatment ends) and at the final follow-up session (6 months after treatment ends), or within two weeks of your final session if you are unable to complete the treatment sessions.

10. Can you leave the study early?

- You can agree to be in the study now and change your mind later.
- If you wish to stop, please tell us right away.
- Leaving this study early will not stop you from getting regular medical care.

If you leave the study early, Johns Hopkins may use or share your health information that it has already collected if the information is needed for this study or any follow-up activities.

11. Why might we take you out of the study early?

You may be taken out of the study if:

- Staying in the study would be harmful.
- You need treatment not allowed in the study.
- You fail to follow instructions.
- The study is cancelled.
- There may be other reasons to take you out of the study that we do not know at this time.

If you are taken out of the study early, Johns Hopkins may use or give out your health information that it has already collected if the information is needed for this study or any follow-up activities.

12. How will your privacy be maintained and how will the confidentiality of your data be protected?

HIPAA Authorization for Disclosure of Protected Health Information

What information is being collected, used, or shared?
To do this research, we will need to collect, use, and share your private health information. By signing this document, you agree that your health care providers (including both Johns Hopkins Medicine and others) may release your private health information to us, and that we may use any and all of your information that the study team believes it needs to conduct the study. Your private information may include things learned from the procedures described in this consent form, as well as information from your medical record (which may include information such as HIV status, drug, alcohol or STD treatment, genetic test results, or mental health treatment).

Who will see, use or share the information?
The people who may request, receive, or use your private health information include the researchers and their staff who may be a part of Johns Hopkins Health System, Johns Hopkins University or the Johns Hopkins Applied Physics Laboratory. Additionally, we may share your information with other people at Johns Hopkins, for example if needed for your clinical care or study oversight. To improve coordination of your research and clinical care, some information about the study you join will be included in your electronic medical record.

By signing this form, you give permission to the research team to share your information with others outside of Johns Hopkins. This may include the sponsor of the study and its agents or contractors, outside providers, study safety monitors, government agencies, other sites in the study, data managers and other agents and contractors used by the study team.

We try to make sure that everyone who sees your information keeps it confidential, but we cannot guarantee that your information will not be shared with others. If your information is disclosed by your health care providers or the research team to others, federal and state confidentiality laws may no longer protect it.

Do you have to sign this Authorization?
You do not have to sign this Authorization, but if you do not, you may not join the study.

How long will your information be used or shared?
Your Authorization for the collection, use, and sharing of your information does not expire. Additionally, you agree that your information may be used for similar or related future research studies.

What if you change your mind?
You may change your mind and cancel this Authorization at any time. If you cancel, you must contact the Principal Investigator in writing to let them know by using the contact information provided in this consent form. Your cancellation will not affect information already collected in the study, or information that has already been shared with others before you cancelled your authorization.

How will your information be protected?
Evaluation and treatment data, MRI images, and video recordings will be stored on secure password protected JHU lab servers and JHU one drive. All are secure cloud-based platforms that are fully HIPAA compliant. Only the PI, co-PIs and authorized research staff will have access to the data.

13. Will the study require any of your other health care providers to share your health information with the researchers of this study?

As a part of this study, the researchers may ask to see your health care records from your other health care providers.

14. What is a Certificate of Confidentiality?

Your study information is protected by a Certificate of Confidentiality. This Certificate allows us, in some cases, to refuse to give out your information even if requested using legal means.

It does not protect information that we have to report by law, such as child abuse or some infectious diseases. The Certificate does not prevent us from disclosing your information if we learn of possible harm to yourself or others, or if you need medical help.

Disclosures that you consent to in this document are not protected. This includes putting research data in the medical record or sharing research data for this study or future research. Disclosures that you make yourself are also not protected.

15. What treatment costs will be paid if you are injured in this study?

Johns Hopkins and the federal government do not have programs to pay you if you are hurt or have other bad results from being in the study. However, medical care at Johns Hopkins is open to you as it is to all sick or injured people.

The costs for any treatment or hospital care you receive as the result of a study-related injury that are not covered by a health insurer will be billed to you.

By signing this form, you will not give up any rights you have to seek compensation for injury.

16. What other things should you know about this research study?

During the study, we will tell you if we learn any new information that might affect whether you wish to continue to participate.

A description of this clinical trial will be available on http://www.ClinicalTrials.gov, as required by U.S. Law. This Web site will not include information that can identify you. At most, the Web site will include a summary of the results. You can search this Web site at any time. If you would like to review the information for this study, or a summary of the results, ask the study team doctor for the ClinicalTrials.gov study registration number.

During this study, you will not have access to certain medical information and test results collected for study purposes. If an emergency occurs while you are in the study, medical information needed for your treatment can be made available to your study doctor and other physicians who treat you. When the study is completed, all the information in your medical record will be available to you.

What is the Institutional Review Board (IRB) and how does it protect you?

This study has been reviewed by an Institutional Review Board (IRB), a group of people that reviews human research studies. The IRB can help you if you have questions about your rights as a research participant or if you have other questions, concerns or complaints about this research study. You may contact the IRB at 410-502-2092 or jhmeirb@jhmi.edu.

What should you do if you have questions about the study, or are injured or ill as a result of being in this study?
Call the principal investigator Dr. Rajani Sebastian at 410-502-5012. If you wish, you may contact the principal investigator by letter. The address is on page one of this consent form. If you cannot reach the principal investigator or wish to talk to someone else, call the IRB office at 410-502-2092.

If you have an urgent medical problem or think you are injured or ill because of this study, call 911 or go to your local emergency department. You should also call Dr. Rajani Sebastian at 410-502-5012 during regular office hours and at 512-207-0280 after hours and on weekends. If this doctor is not available, the operator will page the "on call physician."

17. Optional Study Components:

This part of the consent form is about optional component(s) of the study that you can choose to take part in or not. You can still take part in the main study even if you say "no" to this/these optional component(s).

<u>**Future Contact**</u>
We would like your permission for our research team to contact you in the future. Please note that your decision below does not prevent other researchers at Johns Hopkins from contacting you about other research.

Please sign and date your choice below:

YES ☐ ______________________________ ________________
Signature of Participant Date

NO ☐ ______________________________ ________________
Signature of Participant Date

18. What does your signature on this consent form mean?

Your signature on this form means that you have reviewed the information in this form, you have had a chance to ask questions, and you agree to join the study. You will not give up any legal rights by signing this consent form.

WE WILL GIVE YOU A COPY OF THIS SIGNED AND DATED CONSENT FORM

Signature of Participant	(Print Name)	Date/Time

Signature of Person Obtaining Consent	(Print Name)	Date/Time

Signature of Legally Authorized Representative (LAR)<br>**FOR ADULTS NOT CAPABLE of GIVING CONSENT**	(Print Name)	Date/Time

Relationship of LAR to Participant<br>(Indicate why the LAR is authorized to act as a surrogate health care decision-maker under state or applicable local law)	Date/Time

I have received the separate Insurance and Research Participant Financial Responsibility Information Sheet.

Signature of Participant, LAR or Parent/Guardian	(Print Name)	Date/Time

NOTE: A COPY OF THE SIGNED, DATED CONSENT FORM MUST BE KEPT BY THE PRINCIPAL INVESTIGATOR AND A COPY MUST BE GIVEN TO THE PARTICIPANT. IF APPROPRIATE FOR THIS STUDY, A SCANNED COPY OF THE SIGNED CONSENT FORM SHOULD BE UPLOADED TO THE PARTICIPANT'S EPIC/EMR RECORD (UNLESS NO MEDICAL RECORD EXISTS OR WILL BE CREATED).
